# Population Structure of a Widespread Species under Balancing Selection: The Case of *Arbutus unedo* L.

**DOI:** 10.3389/fpls.2015.01264

**Published:** 2016-01-13

**Authors:** Xabier Santiso, Lua Lopez, Rubén Retuerto, Rodolfo Barreiro

**Affiliations:** ^1^Facultad de Biología, Área de Ecología, Universidade de Santiago de CompostelaSantiago de Compostela, Spain; ^2^Facultad de Ciencias, Área de Ecología, Universidade da CoruñaA Coruña, Spain; ^3^Department of Plant Systematics and Biodiversity, Center for Organismal Studies Heidelberg, University of HeidelbergHeidelberg, Germany

**Keywords:** AFLP, Atlantic-Mediterranean split, *Arbutus unedo*, disjunct distribution, genetic diversity, genetic structure, stabilizing selection

## Abstract

*Arbutus unedo* L. is an evergreen shrub with a circum-Mediterranean distribution that also reaches the Eurosiberian region in northern Iberia, Atlantic France, and a disjunct population in southern Ireland. Due to the variety of climatic conditions across its distribution range, the populations of *A. unedo* were expected to display local adaptation. Conversely, common garden experiments revealed that diverse genotypes from a range of provenances produce similar phenotypes through adaptive plasticity, suggesting the action of stabilizing selection across its climatically heterogeneous range. Nonetheless, since a uniform response might also result from extensive gene flow, we have inferred the population structure of *A. unedo* and assessed whether its extended and largely one-dimensional range influences gene flow with the help of AFLP genotypes for 491 individuals from 19 populations covering the whole range of the species. As we had anticipated, gene flow is restricted in *A. unedo*, providing further support to the hypothesis that stabilizing selection is the most likely explanation for the homogeneous phenotypes along the range. The Euro-Siberian populations were not particularly isolated from the Mediterranean. Instead, there was a distinct genetic divide between the populations around the Mediterranean Sea and those sampled along Atlantic coasts from northern Africa up to Ireland. This genetic structure suggests the action of historic rather than biogeographic factors as it seems consistent with a scenario of independent glacial refugia in the Atlantic and Mediterranean portions of the range of *A. unedo*. Genetic exchange was likewise restricted within each set of populations. Nevertheless, isolation-by-distance (IBD) was stronger, and *F*_ST_ increased faster with distance, along the Atlantic, suggesting that gene flow might be larger among Mediterranean populations. Genetic diversity was significantly lower in NW Iberia and Ireland than in other populations whereas Ireland was more closely related to populations in NW Iberia than to a population in Atlantic France, suggesting a postglacial stepping-stone colonization of the Atlantic coast. Altogether, our results show that stabilizing selection is able to homogenize the phenotypic response even when population structure is strong, gene flow is constrained, and the phylogeographic past is complex.

## Introduction

The future of a species, in terms of adaptive ability and evolutionary potential, depends on its genetic diversity and quantitative traits. These can be affected by the spatial distribution of the species as it influences the amount of gene flow among populations (Frankham et al., 2002). Species with peculiar distributions offer an interesting opportunity to investigate the structure of their genetic and quantitative traits, together with the processes that may have determined it. Plant species with large distributional ranges across contrasting environmental conditions are expected to experience selective pressures. Under these circumstances species can show two non-exclusive strategies. First, species can develop phenotypic adaptations to the local environment, generating ecotypic differentiation in relevant functional traits when growing in their home location (Kawecki and Ebert, 2004; Savolainen et al., 2007). This pattern is commonly observed in locally adapted species where individuals will exhibit higher fitness at their home location (Kawecki and Ebert, 2004; Leimu and Fischer, 2008). Local adaptation is expected to limit effective gene flow and increase genetic differentiation among populations (Kawecki and Ebert, 2004). Nevertheless, other processes such as genetic drift or bottlenecks may likewise limit gene flow and result in a large genetic and quantitative variation among populations; in these cases, variation is not linked to a higher fitness at home locations (Lopez et al., 2015). Second, species can also react to environmental variability by displaying phenotypic plasticity in their traits (Lortie and Aarssen, 1996; Price et al., 2003). In this regard, plasticity has been suggested as an adaptive mechanism (i.e., adaptive plasticity) in response to environmental pressures which allow plants to cope with contrasting conditions (Ghalambor et al., 2007; Palacio-López et al., 2015). However, phenotypic plasticity can also result from extensive gene flow among populations, and plasticity may even hinder local adaptation as long as there is enough migration among populations (Sultan and Spencer, 2002). Whether plasticity is related to ecological selection or to migration cannot be inferred from phenotypic studies alone. In this regard, molecular markers are the proper tools to disentangle the spatial and demographic patterns of genetic variability in a species. An important limitation in non-model organisms is developing molecular markers with appropriate resolving power when little or no sequence information is available. Under these circumstances, amplified fragment length polymorphisms (AFLPs) (Vos et al., 1995) have been widely used in plants because they provide high reproducibility, allow a comprehensive scan of the genome, and do not require previous knowledge of the genome (Mba and Tohme, 2005; Peters et al., 2009; Lopez and Barreiro, 2013). Unfortunately, when compared to other markers (e.g., SSR), AFLP are dominant markers preventing the identification of heterozygotes. Dominant markers also tend to detect lower levels of diversity within population and higher levels of differentiation among populations than their codominant counterparts (Nybom, 2004). Despite the latter, AFLP loci have been shown to be more efficient that SSR loci in systems characterized by weak population structure (Campbell et al., 2003).

**Figure 1:**
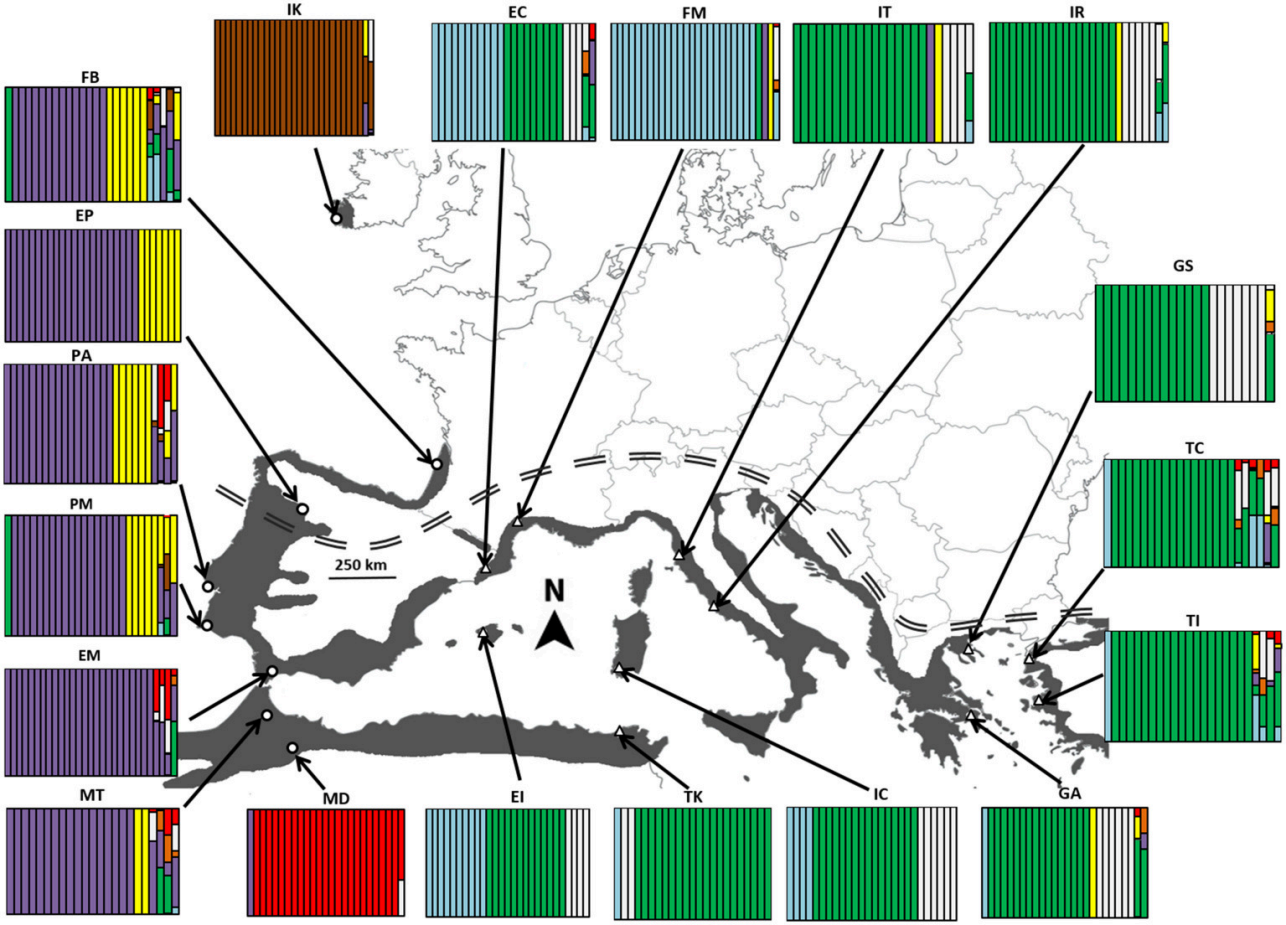
**Distribution range with the location of the 19 *Arbutus unedo* populations sampled in this study and their genetic structure according to an individual-based analysis with BAPS**. Population histograms show the genetic cluster of each individual (vertical bars) for the optimal number of seven genetic clusters (*P* = 0.9793) obtained after admixture with the spatial analysis option. Open triangles and circles are populations assigned to Mediterranean and Atlantic genetic clades, respectively. Double-dashed line indicates the limits between the Euro-Siberian and Mediterranean biogeographical regions. See Materials and Methods for population names.

*Arbutus unedo* L. (strawberry tree) is a predominantly Mediterranean evergreen shrub or small tree (Sealy, 1949; Webb, 1983; Cox and Moore, 2005). The strawberry tree is a typical element of the Mediterranean biogeographical region where it shows a neat circum-Mediterranean distribution (Torres et al., 2002) that evokes the one-dimensional spatial pattern typically found in ring species (Irwin et al., 2001). This small tree occupies a narrow coastal fringe from Tunisia to Morocco along the north of Africa, and from Spain to Turkey along southern Europe (Figure 1). The plant is also widespread in western Iberia and northern Morocco, where the Mediterranean biogeographic region meets the Atlantic Ocean. From there, the strawberry tree spans northwards along Atlantic Europe, entering into the Euro-Siberian region up to the 4°C limit for the mean temperature of January. Thus, it is found in northern Iberia, western France, and there is a disjunct population in southwestern Ireland (Sealy, 1949). The presence of *A. unedo* in Ireland but not in Britain means that this tree belongs to the puzzling Lusitanian flora: organisms that are found in southern and western Ireland and in northern Iberia but are absent from intervening countries (Sealy, 1949; Beatty and Provan, 2013). It has been suggested that the presence of *A. unedo* in Ireland and its absence in Britain might be due to a postglacial migration directly from Iberia (Reid, 1913). Nonetheless, no solid evidence exists for an Iberian origin for the Irish demes, and it was recently hypothesized that *A. unedo* may have arrived in Ireland from the Atlantic coast of France (Cox and Moore, 2005). Despite this interesting distribution, no study has investigated the genetic structure of *A. unedo* throughout its range. This information is essential for accurately interpreting the finding that plants collected along the species range respond similarly to changes in nutrient and water availability when placed in common gardens (Santiso, 2015; Santiso and Retuerto, 2015; Santiso et al., 2015). The low Q_ST_ estimates obtained for traits that are known to be highly affected by climatic factors (e.g., temperature, rain) were interpreted as evidence that the populations of the strawberry tree may be under stabilizing selection despite its extended and climatically variable range (Santiso et al., 2015). Nonetheless, the absence of local adaptation might also indicate that gene flow is large across the range. Studies of the genetic structure of *A. unedo* available so far covered only a very small portion of the species range and produced contrasting results. For example, within-population diversity was determined to be low (Takrouni and Boussaid, 2010), moderate (Lopes et al., 2012) or high (Takrouni et al., 2012) depending on the set of sampled populations. Likewise, genetic differentiation was found to be low (Takrouni et al., 2012) and moderate (Takrouni and Boussaid, 2010) among Tunisian populations, while low-moderate differentiation was recorded among populations in Portugal (Lopes et al., 2012). Also, there is no information on whether the Eurosiberian populations of *A. unedo* show any sign of reduced gene flow with those located in the main Mediterranean range. Similarly, since *A. unedo* occupies most of the shoreline around the Mediterranean but it is largely absent from Syria to Libya (>2500 km), it could be speculated that the populations that encircle the Mediterranean might fit an isolation-by-distance (IBD) pattern if gene flow is restricted to movements along the shoreline. Finally, it would be reasonable to anticipate that the disjunct Irish enclaves may be genetically eroded given their peripheral, isolated position (Vucetich and Waite, 2003).

Here, we developed and applied a set of AFLP markers with high resolving power to obtain a comprehensive image of the population structure of this small tree. In particular, we addressed a number of specific issues: (i) whether the largely one-dimensional distribution of the strawberry tree has restricted gene flow; (ii) whether the climatic contrast between Eurosiberian and Mediterranean populations has reduced the genetic exchange between them; and, in an effort to contribute to the Lusitanian debate, (iii) whether the Irish population is more closely related to French or Iberian ones. To fulfill these aims, we conducted a range-wide survey of *A. unedo* from Turkey in the East to Iberia in the West, and from Morocco in the South to Ireland in the North.

## Materials and methods

### The species

*Arbutus unedo* is a diploid species (2*n* = 26, × = 13) (Sealy and Webb, 1950) belonging to the family Ericaceae, subfamily Arbutoideae, which includes evergreen, shrub-like woody taxa with sclerophyllous, laurel-like leaves (Torres et al., 2002). Genus *Arbutus* has several members in America and four in Europe: *A. andrachne* L. (eastern Mediterranean), *A. pavarii* Pampanini (Libyan coasts), *A. canariensis* Veill. (Canary Islands) and *A. unedo* L. (Torres et al., 2002). Flowering spans from September to December and it is insect pollinated (Mitchell, 1993); indeed, its nectar and pollen are the main food source for *Bombus terrestris* (Rasmont et al., 2005). Fruits take 12 months to ripen, and seeds are dispersed by animals (mainly birds); seed viability was reported to be about 55% in favorable places while seedling loss was found to exceed 60% (Sealy, 1949).

### Sample collection and DNA extraction

Our sampling scheme covered the entire distribution of the species around the Mediterranean Sea and Atlantic coast, and included populations from contrasting environmental conditions that ranged from wet climates in Ireland or western France to sites with a long summer drought in the Mediterranean (ombrothermic diagrams for the areas sampled in this study can be found in Supplementary Material). Leaf samples were collected from 19 sites covering nine countries (Table 1 and Figure 1): two sites in Turkey (Izmir-TI and Çannakale-TC), two in Greece (Athens-GA, Sithonia-GS), one in Tunisia (Kroumerie-TK), three in Italy (Rome-IR, Toscana-IT, Cagliari-IC), two in Morocco (Tanger-MT, Debdou-MD), two in Portugal (Arrabida-PA, Monchique-PM), four in Spain (Catalonia-EC, Mallorca Island-EI, Málaga-EM, Ponferrada-EP), two in France (Montpellier-FM, Bordeaux-FB), and one in Ireland (Killarney-IK). At each population, leaves were collected from 24 to 30 haphazardly selected trees and the final number of available samples was 491. To minimize the risk of sampling half siblings, distance between sampled trees was >10 meters; in this regard, it is worth noting that there is no vegetative reproduction by suckers in *A. unedo* (Sealy, 1949). In the field, leaves were individually wrapped in Kimtech Science wipes (Kimberly-Clark Europe Ltd, United Kingdom) and stored in silica gel until DNA extraction.

**Table 1:** **Sampling sites and genetic diversity estimates based on 125 segregating loci (5% criterion) obtained with AFLPs for *Arbutus unedo***.

**Population**	**Coordinates (UTM)**	***N***	**PL**	**PB**	***H*j (±S.E.)**
IK	29U 465211 5763297	27 (29)	94 (75.2%)	0	0.26 (±0.016)
FB	30T 643276 4939413	24 (26)	103 (82.4%)	0	0.31 (±0.015)
EP	29T 705440 4722721	27 (29)	99 (79.2%)	0	0.28 (±0.016)
PA	29S 497741 4257559	24 (27)	108 (86.4%)	0	0.32 (±0.015)
PM	29S 538873 4126983	24 (27)	109 (87.2%)	0	0.30 (±0.015)
EM	30S 376766 4076446	26 (29)	103 (82.4%)	0	0.30 (±0.015)
MT	30S 267001 3940810	21 (23)	112 (89.6%)	0	0.32 (±0.014)
MD	30S 492658 3759875	23 (25)	108 (86.4%)	0	0.31 (±0.014)
*ATL*		*202 (215)*	*111 (88.8%)*	*0*	*0.31 (± 0.014)*
EC	31T 454080 4620578	24 (25)	108 (86.4%)	0	0.31 (±0.015)
FM	31T 531757 4829416	26 (28)	102 (81.6%)	0	0.30 (±0.015)
EI	31S 461243 4392754	25 (27)	104 (83.2%)	0	0.31 (±0.015)
IC	32S 491121 4333556	24 (26)	114 (91.2%)	0	0.34 (±0.014)
TK	32S 470929 4061547	21 (23)	110 (88.0%)	0	0.32 (±0.015)
IR	33T 279753 4619932	26 (27)	104 (83.2%)	0	0.32 (±0.015)
IT	32T 637192 4811775	21 (23)	109 (87.2%)	0	0.33 (±0.015)
GA	34S 746586 4206313	24 (26)	113 (90.4%)	0	0.34 (±0.014)
GS	34T 739962 4452294	20 (22)	108 (86.4%)	0	0.33 (±0.015)
TC	35T 459279 4441142	23 (24)	107 (85.6%)	0	0.31 (±0.016)
TI	35S 458841 4254352	24 (25)	109 (87.2%)	0	0.32 (±0.015)
*MED*		*263 (276)*	*113 (90.4%)*	*2*	*0.34 (±0.014)*

ATL and MED are total values for populations assigned to Atlantic (8) and Mediterranean (11) genetic lineages by other analyses. See Materials and Methods for population names. Pop, population; N, sample size (effective sample size after accounting for loci with missing values in some individuals); PL, number (and percentage) of polymorphic loci (5% criterion); PB, no. of private bands; Hj, Nei's gene diversity (± standard error).

DNA was extracted with the “Realpure Genomic DNA extraction from plants and fungi kit” (REAL, Durviz s.l.u., Spain) following manufacturer's instructions with minor modifications: incubation times at cell lysis and protein precipitation steps were extended up to 2 h and up to 20 min, respectively. Samples were processed in batches of 23 samples (from at least five populations) plus 1–2 negative controls with no tissue. Ten percent of the samples were extracted twice on different batches. The quality of the extracted DNA and negative controls was checked on 1.5% agarose gels.

### AFLP analyses

Given that AFLP performance can be sensitive to reaction conditions (Bonin et al., 2004), we used several control measures to guarantee the reproducibility of our set of markers. First, primer combinations were chosen after screening 12 pairs of primers, with three selective bases, on 20 individuals from five populations that covered the whole range of the strawberry tree (four individuals per population). The whole procedure was repeated with new, independent DNA extractions of the same 20 individuals to assess the reproducibility of each primer combination. Nine selective primer combinations that were highly reproducible and easy to score were selected (EcoRI/TruI): TGG/CAA TCA/CAT TAG/CAT TCA/CTG TAC/CTG TAG/CTT TGC/CAC TAC/CAA TAG/CTG. Second, DNA was re-extracted from approximately 10% of individuals (evenly distributed among the 19 sampling sites) and run in parallel with other DNA samples to check for reproducibility during the study. Samples and replicates were run in a blind manner to avoid any bias during scoring. In addition, individuals from each one of the 19 sites were evenly partitioned among the 96-well plates used for PCR; replicates and originals were always run on separate plates to avoid potential biases inherent to one particular plate. Third, each batch of DNA extractions (24 samples) included a negative control that went through the entire genotyping procedure (DNA extraction included). The estimated global genotyping error (1.5%) was consistent with results of reproducibility tests conducted for AFLP on both plants and animals (Bonin et al., 2004); the maximum error rate for individual loci (5%) was well below the maximum recommendable for AFLP analyses (10%) (Bonin et al., 2007).

AFLP analyses were performed following Vos et al. (1995) but nonradioactive fluorescent dye-labeled primers were used and fragments were separated on a DNA sequencer. Approximately 250 ng of genomic DNA were digested at 37°C for 3 h in a final volume of 20 μl with 1.25 units of EcoRI and TruI (Fermentas) and 2X Tango Buffer (Fermentas). Digested DNA was ligated for 3 h at 37°C to double-stranded adapters (50 pmols of adaptors E, 5′-CTCGTAGACTGCGTACC-3′ and 5′-AATTGGTACGCAGTCTAC-3′; and M, 5′-GACGATGAGTCCTGAG-3′ and 5′-TACTCAGGACTCAT-3′) using 0.5 units of T4 DNA ligase (Fermentas). Then, 2 μl of the ligation product was pre-amplified with 0.3 μM of each single selective primer (EcoRI-T and TruI-C), 2.5 mM MgCl2, PCR buffer 1X (Applied Biosystems), 0.8 μM dNTPs, 0.04 μg/μl BSA, Betaine 1 M and 0.4 U of Taq polymerase (Applied Biosystems) in a final volume of 20 μl. Amplification conditions were 2 min at 72°C; 2 min at 94°C; 20 cycles of 30 s at 94°C, 30 s at 56°C, and 2 min at 72°C; and a final extension of 30 min at 60°C. Pre-amplification fragments were diluted 1:5 with Milli-Q water; 2.5 μl of the resulting solution were selectively amplified using 0.6 μM of the selective primers, 0.8 μM dNTPS, 2.5 mM MgCl2, 0.04 μg/μl BSA, PCR Buffer 1X (Applied Biosystems) and 0.4 U of AmpliTaq Gold polymerase (Applied Biosystems) in a final volume of 10 μl. Selective amplification was performed as follows: 4 min at 95°C; 12 of cycles of 30 s at 94°C, 30 s at 65°C (first cycle, then decreasing 0.7°C for each one of the last 11 cycles), and 2 min at 72°C; 29 cycles of 30 s at 94°C, 30 s at 56°C, and 2 min at 72°C; and a final extension of 30 min at 72°C. Digestion, ligation, and PCR reactions were performed in a PxE thermal cycler (Thermo Fisher Scientific Inc., Waltham, MA, USA). Selective amplification products were electrophoresed on an ABI 3130xl automated DNA (Applied Biosystems) sequencer with HD-500 as size standard (Applied Biosystems). Fragments from 70 to 400 bp were manually scored for presence/absence at each selected locus with the help of GeneMarker v.1.70 (SoftGenetics LLC, State College, PA, USA) following common recommendations (Bonin et al., 2005).

### Data analysis

Sampling sites were regarded as separate populations and their allele frequencies were estimated using the Bayesian Method of Zhivotovsky (1999) implemented in AFLPsurv 1.0 (Vekemans et al., 2002) with the option of non-uniform prior distributions of allele frequencies. Genetic diversity per population was estimated as the proportion of polymorphic loci (5% criterion), the number of private bands, and Nei's gene diversity (Hj). Significant differences in gene diversity between populations were tested with the GT2-method for multiple unplanned comparisons among pairs of means based on unequal sample sizes (Sokal and Rohlf, 1995).

Allele frequencies were also employed to estimate genetic differentiation between individuals as *F*_ST_ values following Lynch and Milligan (1994). Significance of pairwise *F*_ST_ values was tested by resampling statistics with 10000 permutations. Pairwise *F*_ST_ values were also used to produce ordinations of the populations using non-metric multidimensional scaling (nMDS) (Cox and Cox, 1994) with the help of the statistical package Primer v 6 (PRIMER-E, United Kingdom) (Clarke and Gorley, 2006). To facilitate comparison with other studies, we also estimated the differentiation between populations with an analysis of molecular variance (AMOVA) (Excoffier et al., 1992) that calculates Φ_PT_ (an analog of *F*_ST_), using the squared Euclidean distance between AFLP phenotypes. The significance of the Φ_PT_values was estimated after 9999 random permutations of individuals among populations performed with GenAlex 6.5 (Peakall and Smouse, 2006). The pattern of genetic differentiation was further investigated with a spatial analysis of molecular variance (SAMOVA) that defines groups of populations that are geographically homogeneous and maximally differentiated from each other. SAMOVA was conducted with the software SAMOVA 1.0 (Dupanloup et al., 2002) running 100 simulated annealing processes for each configuration of *K* groups, with *K* ranging from 2 to 17, and searching for the configuration that maximize *F*_CT_ (the proportion of total genetic variance due to differences among groups of populations).

The correlation between genetic distance (pairwise *F*_ST_ values) and geographic distance (km) among populations was tested with the Mantel test implemented in the IBD Web Service 3.23 (Jensen et al., 2005); significance was tested with 10,000 bootstrap randomizations. Tests for IBD were repeated for straight-line distances and for a matrix of geographic distances estimated avoiding conspicuous barriers to dispersal (high mountains and long sea stretches). We repeat these analysis using the Nei's genetic distance between populations.

An alternative view of the population structure was obtained with the individual-based Bayesian clustering algorithms implemented in BAPS 6.0. Initially, genetic mixture analyses were done using both the spatial (Corander et al., 2008) and the non-spatial model (Corander et al., 2006). However, since both clustering models yielded very similar partitions, only the results of the spatial model are shown here. With the complete data set (19 populations), BAPS was run three times for each *K* from 2 until 25. Later, separate analyses were run for the 11 Mediterranean populations and for the 8 Atlantic populations with *K* from 2 to 15. The optimal partition determined by the program was used to estimate the levels of genetic admixture of individuals with 200 reference individuals simulated for each genetic group and each original individual analyzed 20 times. To assess the strength of the signal in our data, the population structure was confirmed with the alternative Bayesian approach implemented in STRUCTURE v.2.3.3 (Pritchard et al., 2000; Falush et al., 2003). STRUCTURE was run assuming correlated allele frequencies, with a burn-in period of 150,000 replications and a run length of 1,000,000 Markov chain Monte Carlo (MCMC) steps. Four iterations per *K* were performed for a number of clusters ranging from *K* = 1 to *K* = 24 (complete data set of 19 populations), and six iterations per *K* were performed for *K* ranging from 1 to 15 (separate analyses of the Mediterranean and Atlantic groups). The value of *K* that captured most of the structure in our data was determined using the approach originally proposed by Pritchard et al. (2000), with further guidance derived from the procedure of Evanno et al. (2005). Runs of selected *K* were averaged with the CLUMPP version 1.1.2 (Jakobsson and Rosenberg, 2007).

Finally, the occurrence of fine-scale spatial genetic structure (SGS) was investigated with GenAlex 6.5 (Peakall and Smouse, 2006). This procedure calculates an autocorrelation coefficient (*r*) that is closely related to Moran's I (Peakall et al., 2003) and measures the genetic similarity between pairs of individuals whose geographic separation falls within a set of specified distance classes. In our study, distance class size was variable to get a similar number of individuals within each distance class (distance class limits: 15, 25, 40, 70, 100, 150, 250, and 500 m). The occurrence of spatial autocorrelation on each site was assessed with a multiclass test criterion (ω) with null hypothesis of *r* = 0 (Smouse et al., 2008). The significance of ω was estimated with 1000 random permutations and significance was declared when *P* < 0.01 following Banks and Peakall (2012). In addition, to assess whether the various populations showed differences in their fine-scale SGS, an overall test of heterogeneity was calculated and its significance assessed with 1000 bootstrap resamples.

## Results

Nine primer combinations produced a total of 226 reproducible AFLP loci, 125 (55.3%) of which were polymorphic for the whole data set (5% criterion) and were kept for further analyses. The number of polymorphic loci per population ranged from 94 (IK in the northern Atlantic edge of the distribution range) to 114 (IC in the Mediterranean; Table 1). Likewise, Nei's gene diversity ranged from 0.26 ± 0.016 in IK to 0.34 ± 0.014 in IC and GA (two populations in the Mediterranean). The GT2-method showed that Nei's diversity in two Atlantic sites (IK and EP) was significantly lower than in any other population (*P* < 0.05). Moreover, IK was significantly less diverse than EP (0.28 ± 0.016). No population showed private bands.

Since the analyses of population structure revealed two clearly separated sets of populations (see below), separate estimates of diversity were obtained for each group. The Mediterranean group (EC, FM, EI, IC, TK, IR, IT, GA, GS, TC, TI) had slightly more polymorphic loci (113 vs. 111) and significantly higher gene diversity (0.34 ± 0.014 vs. 0.31 ± 0.014, *P* < 0.001, *t*-test) than the Atlantic group (IK, FB, EP, PA, PM, EM, MT, MD). Besides, two private bands were detected in some, but not all, Mediterranean populations.

**Figure 2:**
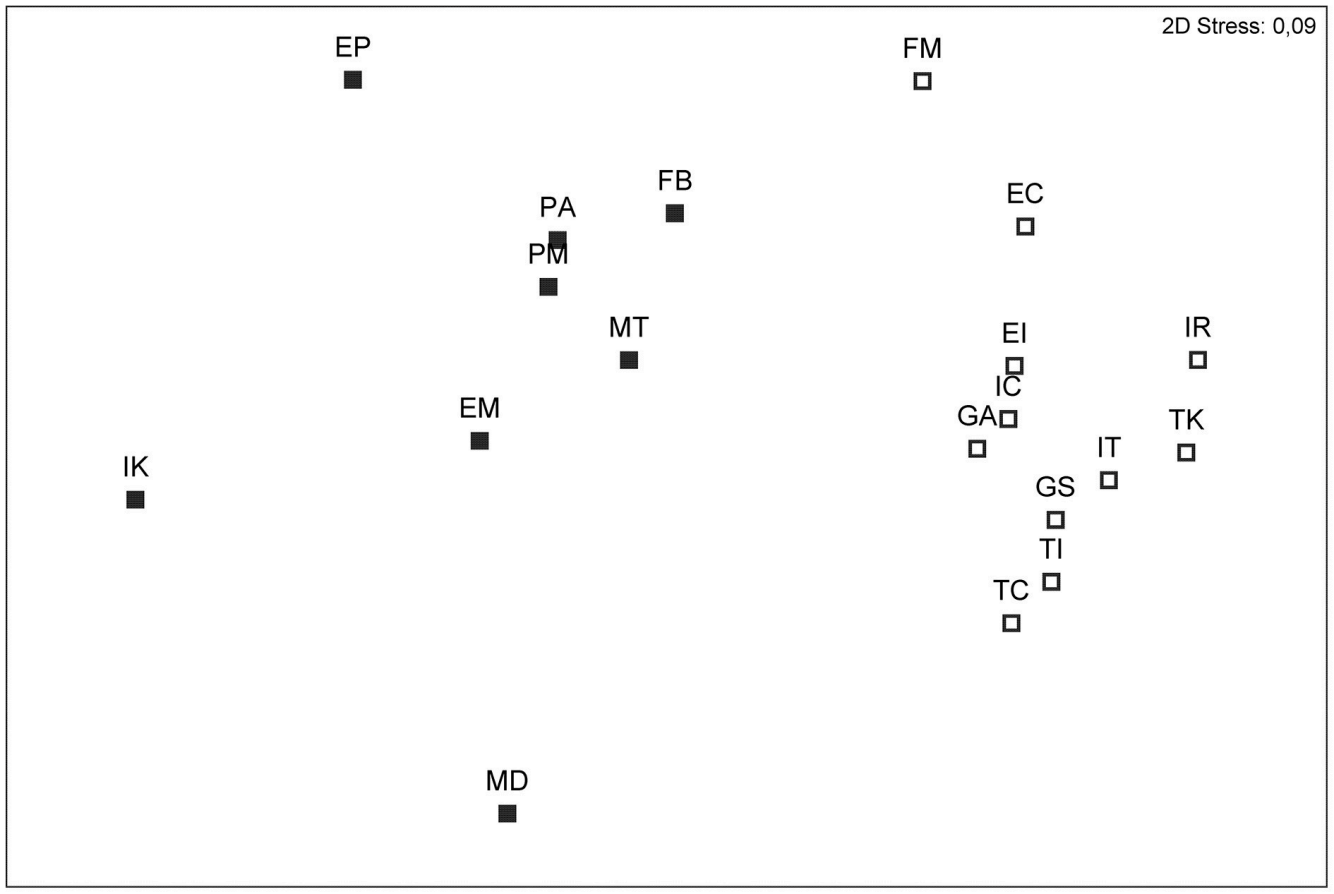
**Non-metric multidimensional scaling (nMDS) showing genetic distances between 19 populations of *Arbutus unedo***. Genetic distances were estimated as pairwise *F*_ST_ values obtained after inferring allele frequencies with the Bayesian method of Zhivotovsky (1999) implemented in AFLP-SURV. Open symbols are populations assigned to the Mediterranean genetic group, closed symbols are populations assigned to the Atlantic group. See Materials and Methods for population names.

Both the global *F*_ST_ (0.083) and Φ_PT_ statistics (0.105) revealed the occurrence of highly significant genetic differences among populations (*P* < 0.001), although most of the variation occurred within populations (90%; Table 2). Pairwise Φ_PT_ estimates were likewise significant (*P* < 0.05), and the pattern of pairwise *F*_ST_ values depicted by the nMDS revealed two sets of populations: the set of circum-Mediterranean sites and the set of demes sampled along the Atlantic coast (Figure 2). The stress coefficient (0.09) indicated that the 2-dimensional plot was a good representation of *F*_ST_ values. Figure 2 showed that the Mediterranean group was more compact than the Atlantic one, suggesting that differentiation was lower among Mediterranean populations. In fact, only FM showed signs of moderate genetic differentiation and its average pairwise *F*_ST_ value (0.069) clearly surpassed the values registered for other Mediterranean sites (average pairwise *F*_ST_ values ranged from 0.028 to 0.049). In comparison, three Atlantic populations visibly separated from the Atlantic set. Interestingly, the three divergent populations included the two sites sampled at the northern (IK) and southern (MD) edges of the distribution range of *A. unedo* along the Atlantic and the site sampled on northwest Iberia (EP). Average pairwise *F*_ST_ values for these three populations ranged from 0.075 to 0.103, clearly exceeding the estimates recorded among other Atlantic sites (0.045–0.062).

**Table 2:** **Analysis of molecular variance (AMOVA) based on 125 segregating AFLPs loci obtained for 491 individuals from 19 populations of *Arbutus unedo* sampled throughout its whole distribution range**.

**Source of variation**	***d.f.***	**MS**	**Variance components**	***P*-values**	**Φ statistics**
**GLOBAL (19 POPULATIONS)**					
Among Populations	18	82.882	2.412 (10%)	<0.0001	Φ_PT_ = 0.105
Within Populations	472	20.583	20.583 (90%)		
**REGIONS (2 REGIONS)**					
Among regions	1	555.995	2.060 (10%)	<0.001	Φ_RT_ = 0.103
Among populations	17	57.409	1.594 (8%)	<0.001	Φ_PR_ = 0.089
Within populations	472	16.262	16.262 (82%)	<0.001	Φ_PT_ = 0.183

Φ_PT_, differentiation among populations; Φ_RT_, differentiation among regions; Φ_PR_, differentiation among populations inside the regions.

SAMOVA results corroborated the partitioning of populations in two sets as well as the presence of a few genetically differentiated populations (Table 3). As expected, *F*_CT_ estimates increased with larger *K* values but reached a plateau for *K* = 4 (*F*_CT_ = 0.125, *P* < 0.001). The optimal partition separated the circum-Mediterranean set from the samples collected along the Atlantic. Most of the Atlantic populations fell together in a single group but IK (northern edge) and MD (southern edge) were held separately. EP separated from other populations for *K* = 5, but the *F*_CT_ estimate for this structure (0.124, *P* < 0.001) indicates that keeping EP as a separate entity does not improve the proportion of genetic variance attributed to differences between groups of populations. Unlike nMDS, SAMOVA provided not support to the hypothesis that FM should be regarded as particularly differentiated from other Mediterranean populations.

**Table 3:** Fixation indices corresponding to the groups of populations (19 in total) of Arbutus unedo inferred by spatial analysis of molecular variance (SAMOVA) algorithms (^***^P < 0.001).

	**Groups composition**	***F*_SC_**	***F*_ST_**	***F*_CT_**
Two groups	1. EM, EP, FB, IK, MD, MT, PA, PM (Atlantic group)	0.091^***^	0.191^***^	0.111^***^
	2. EC, FM, EI, IC, IR, IT, TK, GS, GA, TC, TI (Mediterranean group)			
Three groups	1. IK	0.081^***^	0.189^***^	0.118^***^
	2. EM, EP, FB, MD, MT, PA, PM (Atlantic group)			
	3. EC, FM, EI, IC, IR, IT, TK, GS, GA, TC, TI (Mediterranean group)			
Four groups	1. IK	0.0725^***^	0.188^***^	0.125^***^
	2. MD			
	3. EM, EP, FB, MT, PA, PM (Atlantic group)			
	4. EC, FM, EI, IC, IR, IT, TK, GS, GA, TC, TI (Mediterranean group)			
Five groups	1. IK	0.069^***^	0.184^***^	0.124^***^
	2. MD			
	3. EP			
	4. EM, FB, MT, PA, PM (Atlantic group)			
	5. EC, FM, EI, IC, IR, IT, TK, GS, GA, TC, TI (Mediterranean group)			

F_SC_, genetic differentiation between populations within groups; F_ST_, genetic differentiation between samples; F_CT_, genetic variance due to differences among groups of populations.

**Figure 3:**
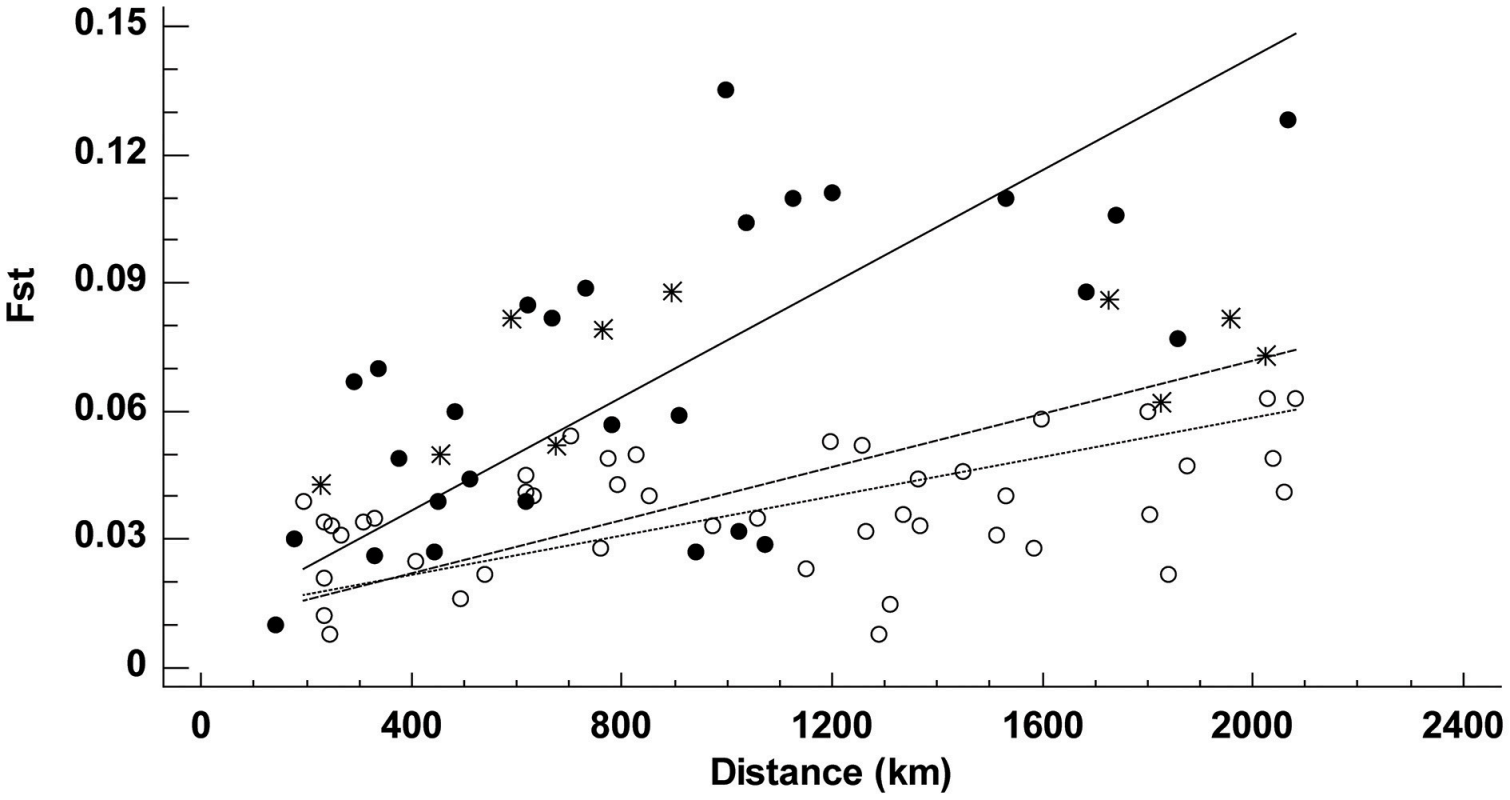
**Relationship between geographic and genetic (*F*_ST_) distances among 19 populations of *Arbutus unedo***. Separated analyses were conducted for Atlantic (solid circles) and Mediterranean (asterisks and open circles) populations; asterisks denote pairwise comparisons with population FM. Lines are reduced major axis regressions for the Atlantic group (solid line, *r* = 0.64, *P* = 0.990), the Mediterranean group (dashed line, *r* = 0.36, *P* = 0.991), and the Mediterranean group with FM excluded (dotted line, *r* = 0.43, *P* = 1.000).

The Mantel test revealed the existence of IBD throughout the entire range of *A. unedo* (19 populations), but the correlation geographic and genetic distance (as *F*_ST_) improved slightly when geographic distance was measured as a straight-line (*r* = 0.57, *P* < 0.001) rather than avoiding conspicuous barriers to dispersal (*r* = 0.48, *P* < 0.001). Separate analyses for each set of populations (straight-line geographic distances only) revealed that both the slope and the strength of the relationship changed from one area to another (Figure 3). The correlation was tighter (*r* = 0.64, *P* = 0.010) and steeper (slope = 6.65 × 10^−5^ ± 1.00 × 10^−5^) in the Atlantic than in the Mediterranean (*r* = 0.36, *P* = 0.009, slope = 3.13 × 10^−5^ ± 4.01 × 10^−6^). Since the highest *F*_ST_ estimates recorded within the Mediterranean were consistently produced by comparison with a single population (FM), Mantel calculations were repeated with FM excluded from the Mediterranean group (Figure 3). Excluding FM improved the correlation (*r* = 0.43, *P* < 0.001) and yielded an even flatter relationship (slope = 2.30 × 10^−5^ ± 3.17 × 10^−6^). Similar results were obtained with Nei's genetic distance between populations instead of *F*_ST_.

The spatial analysis of BAPS identified that the optimal number of genetic clusters (K*)* for the 19 populations was 7 (log-likelihood = −25960.681, probability for 7 clusters = 0.9793); a majority of individuals (91.4%) were assigned to a single genetic cluster. Admixture clustering graphs reinforced the split between Atlantic and Mediterranean because most of the genetic clusters were largely region-specific (Figure 1). Likewise, plants from IK and MD were consistently assigned to separate lineages. The non-spatial analysis with BAPS yielded similar results, suggesting that the use of a geographical prior did not compromised the partition. Results from STRUCTURE (not shown) revealed a similar split between Mediterranean and Atlantic, and corroborated the peculiar genetic composition of IK and MD.

Finally, spatial autocorrelation analyses failed to detect any evidence of fine-scale SGS. The test of heterogeneity among populations indicated that the 19 sites displayed a similar lack of spatial pattern (total ω = 102.867, *P* = 1.000). The combined autocorrelogram was non-significant (multiclass test ω = 23.441, *P* = 0.073) and showed a very flat profile around the null hypothesis of *r* = 0.

## Discussion

As we had anticipated, the range-wide genetic structure of *A. unedo* revealed restrictions to gene flow. Not only was the global Φ_PT_ estimate significant, but each and every pairwise Φ_PT_ comparison among populations was highly significant too. It could be argued that the moderate Φ_PT_ (or *F*_ST_) obtained in our analyses, together with the fact that most of the genetic variation was found within populations, could be regarded as a sign that gene flow might still play a role in the genetic structure of the strawberry tree. Nonetheless, it should be recaled that Φ_PT_ measures are strongly influenced by the amount of within population genetic variation, which in our case were high. Hence, considerable genetic variation observed within the populations of the strawberry tree, rather than gene flow, might possibly explain the moderate Φ_PT_ value detected in our study. Furthermore, a comparable restriction in gene flow was also observed with cpDNA markers while studying the phytogeography of *A. unedo* (Santiso, 2015). The congruence between molecular markers with different modes of inheritance adds further support to the restriction to gene flow in the strawberry tree. Consequently, our results provide further support to the proposal that stabilizing selection and adaptive plasticity, rather than gene flow, seem the mechanisms behind the low Q_ST_ values and homogenous physiological performance observed along the species range (Santiso et al., 2015). Adaptive plasticity has been previously suggested as an evolutionary alternative to local adaptation as moderate levels of phenotypic plasticity are known to be evolutionary advantageous (Price et al., 2003; Kawecki and Ebert, 2004; Palacio-López et al., 2015). Moderate-high levels of genetic variation can act as a major evolutionary mechanism in species with an environmentally heterogeneous range. In this regard, our estimates indicate that *A. unedo* retains high levels of genetic variation within most of its populations. Moreover, phenotype in *A. unedo* seems rather insensitive to genetic erosion and/or isolation because the performance of plants from populations that are genetically depleted and/or have a distinctive genetic composition (IK, EP, MD, FM) was previously shown to be indistinguishable from the behavior found in plants collected throughout the species range (Santiso, 2015; Santiso and Retuerto, 2015).

At the onset of this study, we had also speculated that the circum-Mediterranean range of *A. unedo* evokes the one-dimensional distribution pattern of a ring species (Irwin et al., 2001) and may thus fit a pattern of IBD. Interpopulation variation in *A. unedo* does fit an IBD pattern in the Mediterranean but, unlike the expectations for a ring species, *F*_ST_ was more closely related to geographic distances estimated as straight-lines (i.e. across the Mediterranean) than avoiding barriers (i.e. around the Mediterranean). Consequently, the circum-Mediterranean distribution does not seem to be a determinant of the restricted gene flow suggested by the IBD pattern. Moreover, genetic differentiation was generally lower among circum-Mediterranean populations than among Atlantic populations separated by similar geographic distances, suggesting that gene flow around the Mediterranean, although restricted, has been higher than between Atlantic demes (Templeton, 2006). Nonetheless, the steeper IBD and higher *F*_ST_ estimates recorded along the Atlantic may also be partially attributed to historical events. Ramachandran et al. (2005) showed that a stepping stone colonization model can generate a gradual increase in genetic differentiation with increasing distance from the initial source population. Accordingly, the colonization of the Atlantic coast by a gradual northward expansion following a serial-founder model may have favored a non-equilibrium explanation for the IBD pattern detected in the Atlantic clade (Kimura and Weiss, 1964).

**Figure 4:**
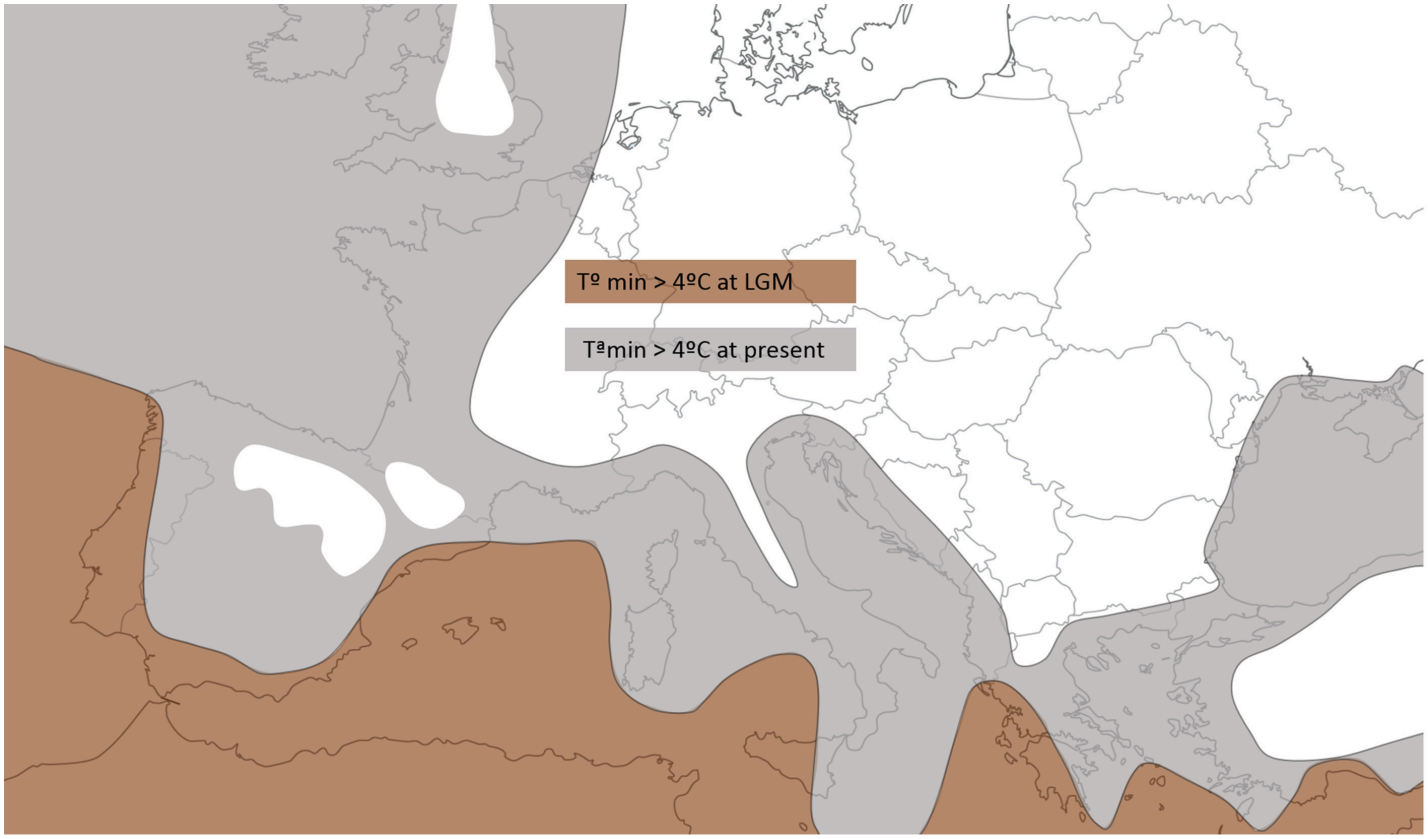
**Area of Western Mediterranean and Atlantic facade with minimum monthly temperatures >4°C (limit for *A. unedo* survival) during the LGM (21 kya, brown) and in modern times (gray) according climate simulation models (Van Andel, 2002 adapted from plots available in http://www.esc.cam.ac.uk/research/research-groups/oistage3/stage-three-project-simulations)**.

A detailed examination of Figure 3 also shows that Atlantic populations separated by less than 1500 km fitted an IBD pattern but non-equilibrium conditions (no IBD) appeared at larger distances. Distances >1500 km always involved comparisons with the Irish deme. In these cases, differentiation no longer depends on geographic distance. Instead, it possibly reflects the effects of the reduced gene flow and larger drift induced by a founder effect (Hutchison and Templeton, 1999), and the distinctive nature of the Irish population is further supported by the individual-based analysis with BAPS (Figure 1). Similarly, the genetic differentiation detected in the Moroccan population MD, at the southern edge of the species range, could be a consequence of fragmentation followed by a reduction in population size (Frankham, 2005). Ireland's colonization may have taken place in postglacial times but earlier than 4000 BP because (i) pollen and charcoal analyses indicate that *A. unedo* has been present in Ireland since at least 4000 BP (Mitchell, 1993; Van Rijn, 2004), and (ii) the earliest plant colonists seemingly arrived to Ireland around 13000 BP but, with the exception of *Betula*, no trees arrived until 9600 BP (Mitchell, 2006). Moreover, paleoclimatic reconstructions show that winter temperature in Ireland during the LGM was clearly below the 4°C limit required for the survival of the strawberry tree (Sealy, 1949; Figure 4). A similar postglacial colonization from southern refugia has been proposed for other Lusitanian elements based on Ecological Niche Model (ENM) reconstructions (Beatty and Provan, 2013, 2014).

Within-population diversity in most of our populations was similar to estimates reported in previous, smaller-scale studies of *A. unedo* and other Ericaceae (Zawko et al., 2001; Takrouni and Boussaid, 2010; Lopes et al., 2012), higher than in the North American species *Arbutus menziesii* (Beland et al., 2005), and in agreement with estimates reported for late-successional perennials with outcrossing breeding system (Nybom, 2004). Nonetheless, we found significant evidence of diminished genetic variation in NW Iberia (11% lower) and Ireland (20%) that seemingly agrees with a stepping-stone colonization from S Iberia to N Iberia and, subsequently, to Ireland. Pairwise *F*_ST_ values also support a connection between Iberia and Ireland because the Irish deme is genetically closer to those from Iberia than to the deme sampled in the French Atlantic. ENM reconstructions for other Lusitanian species also showed the presence of glacial refugia in North Iberia (Beatty and Provan, 2013, 2014). Consequently, the arrival of *A. unedo* to Ireland via the Celtic Sea, as shown by Mitchell (2006) for other tree species, seems more likely than the previously hypothesized route via France, and should be taken into consideration when interpreting possible human-mediated introductions to the island.

From a genetic perspective, the strawberry tree seems segregated in two sets of populations that, unlike our expectations, do not correlate with the biogeographic regions occupied by this small tree (Eurosiberian, Mediterranean). Instead, there is a separation between the populations located around the Mediterranean basin and those found along Atlantic coasts from North Africa up to Ireland. The genetic partition involves two private AFLP loci in the circum-Mediterranean group, suggesting that the split may have taken place long time ago (Vilatersana et al., 2007). The circum-Mediterranean group had slightly, but significantly, higher levels of within-population genetic variation, suggesting a more ancient origin for that set of populations (Rodríguez-Sánchez et al., 2009). Alternatively, the higher diversity recorded in the Mediterranean basin may also have historical roots as sequence data for several fragments of the cpDNA genome suggests that the Mediterranean basin was colonized by two distinct matrilineal lineages (Santiso, 2015).

The Iberian Peninsula appears to be the contact zone between the Mediterranean and Atlantic clades. In Iberia, demes from each group are separated by relatively short distances (<750 km). In comparison, populations within each genetic group (i.e., Mediterranean and Atlantic) can be separated by 2000 km. Several other widespread Iberian trees also show an ancient and often remarkably clear-cut divide between populations from the Mediterranean and from the Atlantic regions of the Iberian Peninsula. The genealogical concordance between multiple co-distributed species can be interpreted as evidence that the responsible evolutionary forces must have had widespread effects at the level of biotic communities and ecosystems (Avise, 2009). In the particular case of Iberian trees, the genetic divide has been interpreted as evidence of mutually isolated glacial refugia located near each of the two coasts, followed by a subsequent expansion inland after the LGM (Benito Garzón et al., 2007; López de Heredia et al., 2007; Médail and Diadema, 2009; Rodríguez-Sánchez et al., 2010). Moreover, a recent review of phylogeographic studies of trees concluded that the genetic divide could even antedate the LGM, arising when species entered Iberia from the south and expanded independently along both coastlines (Rodríguez-Sánchez et al., 2010). Whether this hypothesis may also apply to the case of *A. unedo* requires further research.

In conclusion, our results show clear constraints to gene flow across the range of *A. unedo*, reinforcing the conclusion that the uniform phenotype found in range-wide common gardens must be due to a combination of adaptive plasticity and stabilizing selection, where the latter selects for a norm of reaction that develops a similar phenotype in individuals from different populations (Santiso et al., 2015). Phenotype homogenization has been strong enough to overcome the strong genetic structure and complex demographic past inferred for the strawberry tree. Some of the genetic patterns detected in the strawberry tree agree with what has been observed in other widespread trees (reviewed in Nieto Feliner, 2014), and may be regarded as evidence of an ancient phylogeographic structure (Avise, 2009). In particular, the strawberry tree is clearly segregated in two genetic lineages, Atlantic and Mediterranean, whose likely origin are historical events during the LGM rather than biogeographical factors. Similarly, the postglacial colonization of the Atlantic coast may have follow a stepping-stone model that resulted in a gradual northwards decrease of within-population genetic diversity, specially, in Ireland. Despite these many opportunities to develop locally adapted genotypes, the strawberry tree has instead retained a considerable plasticity that produces a similar phenotype even from diverse genotypes from populations affected by contrasting environments (Santiso, 2015). Altogether, our results indicate that a strong genetic structure, a wide and climatically variable range, and even a complex demographic past, do not necessarily predict the development of local adaptations.

## Author contributions

XS, LL, RR, and RB designed the study. XS and RR collected the data. XS, LL, and RB analyzed the data. XS, LL, RR, and RB interpreted the data. XS drafted the manuscript and LL, RR, and RB critically revised the manuscript. All authors read and approved the final version of the manuscript.

## Funding

This research was supported by research grant CGL2009-11356 (Ministerio de Ciencia e Innovación) and FPU fellowship AP-2009-0962 (Ministerio de Educación). This research was also supported by the European Regional Development's Fund (ERDF).

### Conflict of interest statement

The authors declare that the research was conducted in the absence of any commercial or financial relationships that could be construed as a potential conflict of interest.
