# Supplementary material for: Population Structure of a Widespread Species under Balancing Selection: The Case of Arbutus unedo L
Source: Front Plant Sci. 2016 Jan 13;6:1264. doi: 10.3389/fpls.2015.01264 (PMC4710743; doi:10.3389/fpls.2015.01264)
Supplement: Supplementary file 1 [file Table1.DOCX]

**Table S1.** Ombrothermic (Walter Lieth) diagrams for meteorological stations close to the populations of *Arbutus unedo* sampled for this study. Following the World Meteorological Organization, climate conditions are depicted using data recorded over the 30-year period from 1961 to 1990 (World Meteorological Organization, 2015). Data from weather stations was originally gathered by the World Meteorological Organization (WMO), and then processed by the National Climatic Data Center of the National Oceanic and Atmospheric Administration (National Oceanic and Atmospheric Administration, 2015). For detailed data for each station, follow the appropriate hyperlink.

| Sampling site: IK  Meteorological station:  Valentia Observatory, Ireland  51º56'N 010º15'W, 14 m  <ftp://ftp.atdd.noaa.gov/pub/GCOS/WMO-Normals/RA-VI/IE/03953.TXT> | 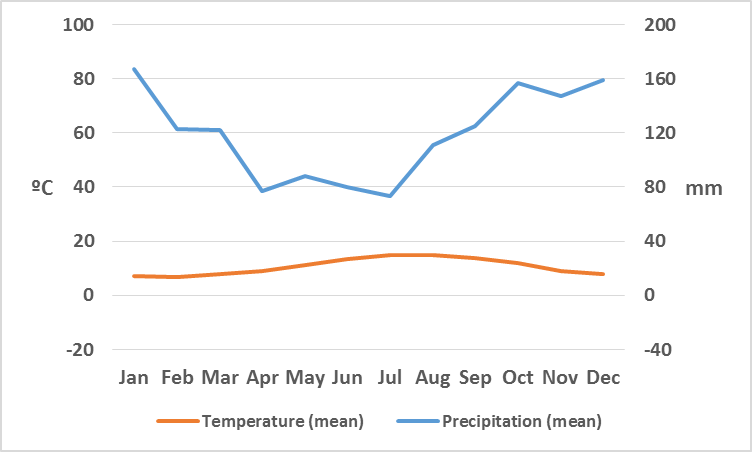 |
| --- | --- |
| Sampling site: FB  Meteorological station:  Bordeaux, France  44º 50' N 000º 42' W, 61 m  <ftp://ftp.atdd.noaa.gov/pub/GCOS/WMO-Normals/RA-VI/FR/07510.TXT> | 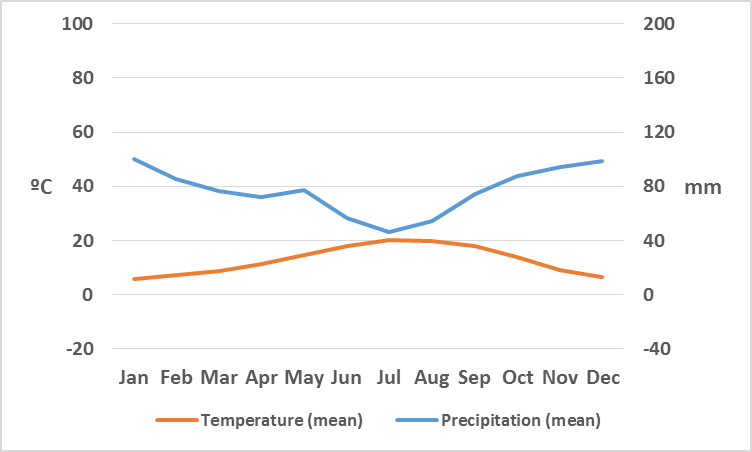 |
| Sampling site: EP  Meteorological station:  Bragança, Portugal  41º 48’ N 6º 44’ W, 692 m  <ftp://ftp.atdd.noaa.gov/pub/GCOS/WMO-Normals/RA-VI/PO/08575.TXT> | 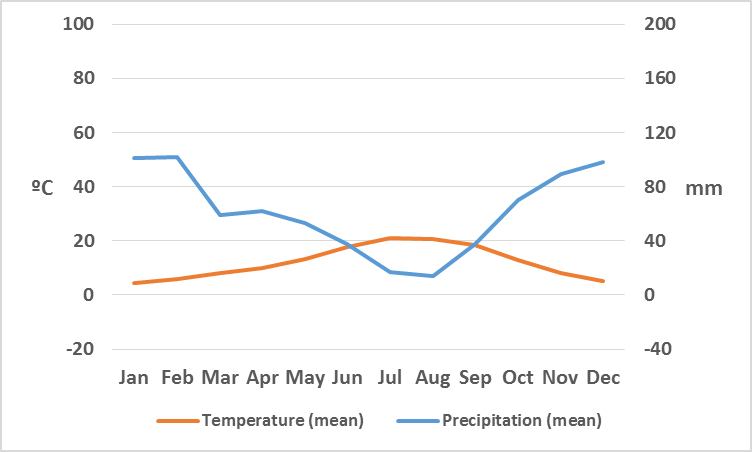 |
| Sampling site: PA  Meteorological station:  Lisboa, Portugal  38º 43' N 009º 09' W, 95 m  <ftp://ftp.atdd.noaa.gov/pub/GCOS/WMO-Normals/RA-VI/PO/08535.TXT> | 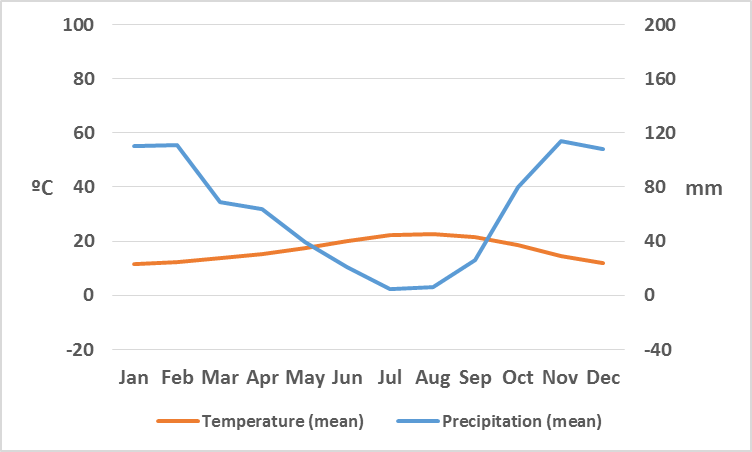 |
| Sampling site: PM  Meteorological station:  Faro, Portugal  37º 01' N 007º 58' W, 8 m  <ftp://ftp.atdd.noaa.gov/pub/GCOS/WMO-Normals/RA-VI/PO/08554.TXT> | 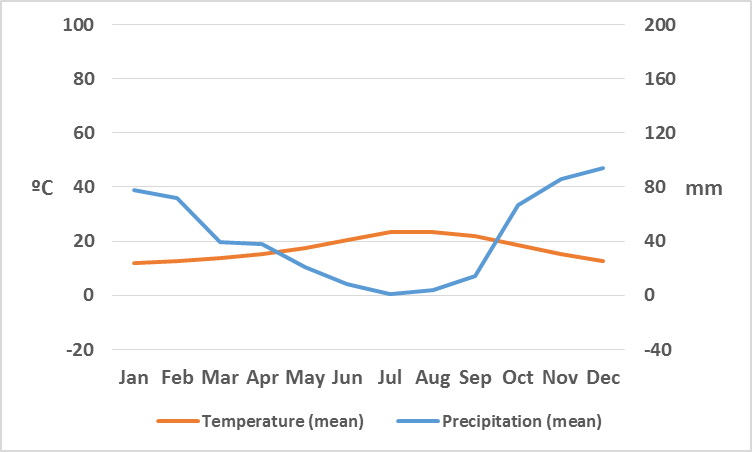 |
| Sampling site: EM  Meteorological station:  Malaga Airport, Spain  36º 40' N 004º 29' W, 7 m  <ftp://ftp.atdd.noaa.gov/pub/GCOS/WMO-Normals/RA-VI/SP/08482.TXT> | 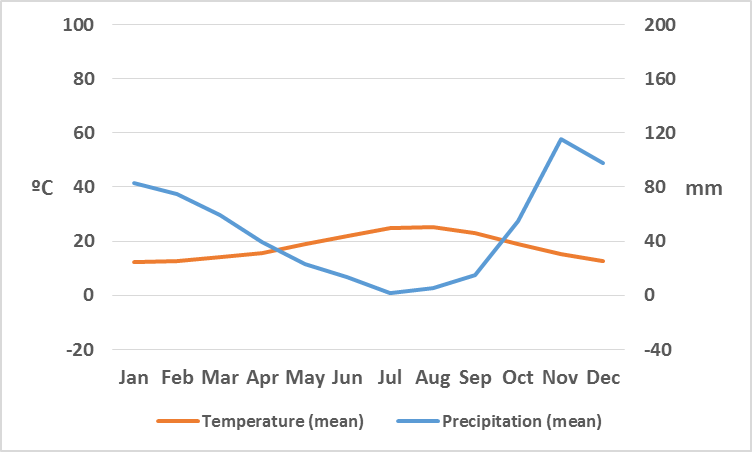 |
| Sampling site: EC  Meteorological station:  Gerona/Costa Brava, Spain  41º 54' N 002º 46' W, 129 m  <ftp://ftp.atdd.noaa.gov/pub/GCOS/WMO-Normals/RA-VI/SP/08184.TXT> | 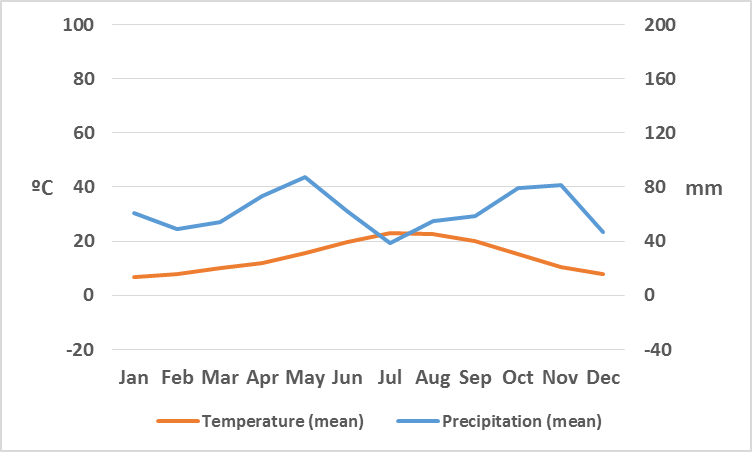 |
| Sampling site: EI  Meteorological station:  Palma de Mallorca, Spain  39º 33' N 002º 44º E, 8 m  <ftp://ftp.atdd.noaa.gov/pub/GCOS/WMO-Normals/RA-VI/SP/08306.TXT> | 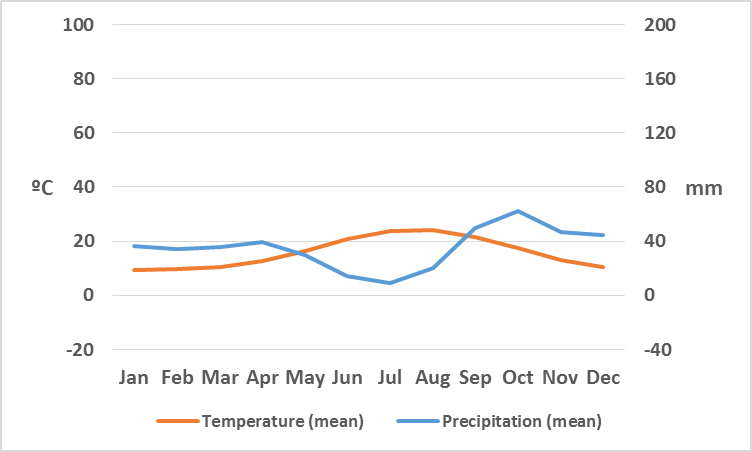 |
| Sampling site: FM  Meteorological station:  Nimes/Courbessac, France  43º 52' N 004º 24' E, 62 m  <ftp://ftp.atdd.noaa.gov/pub/GCOS/WMO-Normals/RA-VI/FR/07645.TXT> | 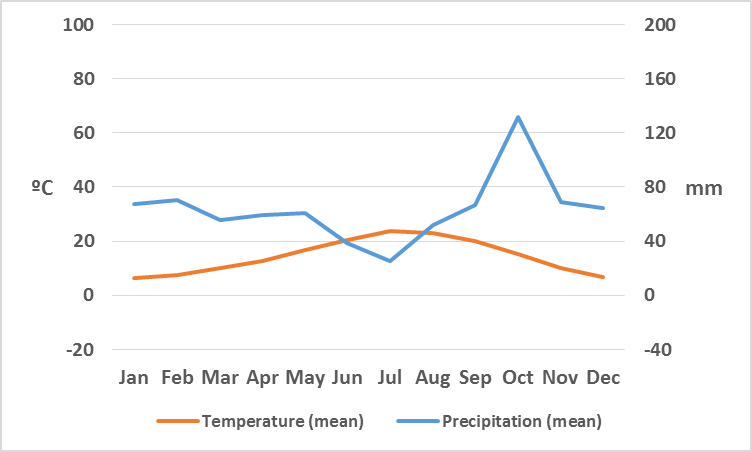 |
| Sampling site: MT  Meteorological station:  Tangier, Morocco  35º 44' N 005º 54' W, 21 m  <ftp://ftp.atdd.noaa.gov/pub/GCOS/WMO-Normals/RA-I/FM/60101.TXT> | 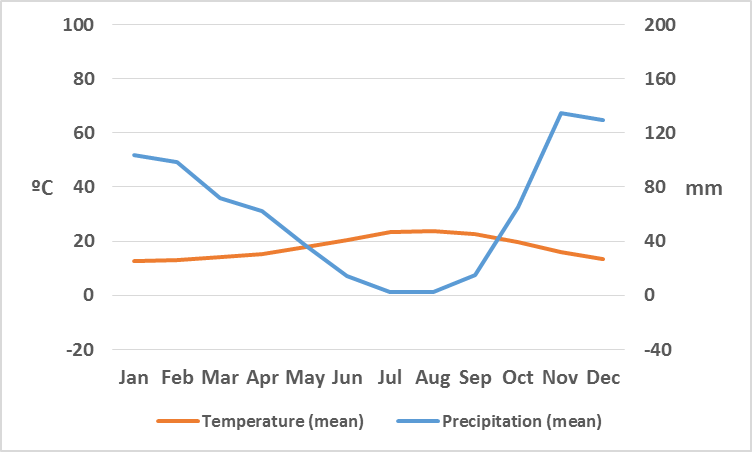 |
| Sampling site: MD  Meteorological station:  Taza, Morocco  34º 13' N 004º 00' W, 510 m  <ftp://ftp.atdd.noaa.gov/pub/GCOS/WMO-Normals/RA-I/FM/60127.TXT> | 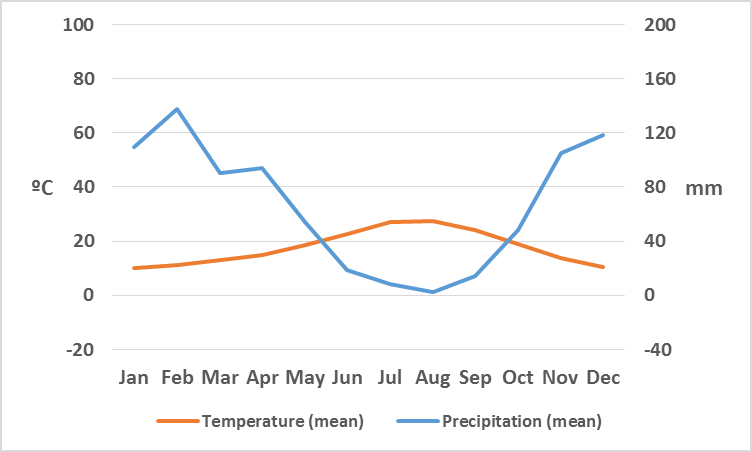 |
| Sampling site: TK  Meteorological station:  Jendouba, Tunisia  36º 29' N 008º 48' E, 143 m  <ftp://ftp.atdd.noaa.gov/pub/GCOS/WMO-Normals/RA-I/TS/60725.TXT> | 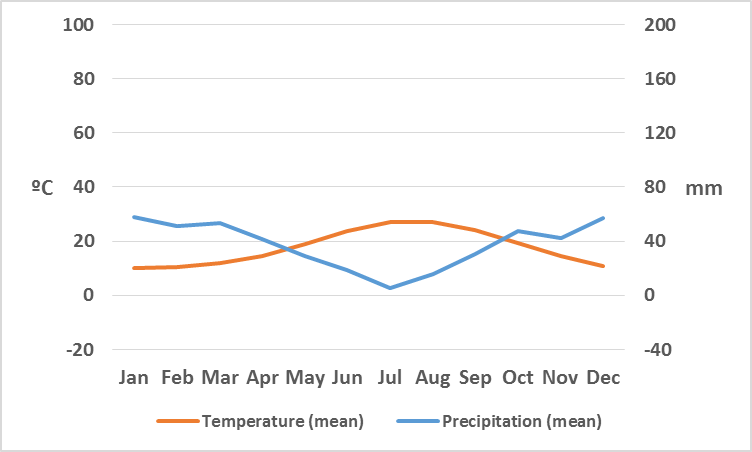 |
| Sampling site: IT  Meteorological station:  Volterra, Italy  43º 24' N 010º 52' E, 575 m  <ftp://ftp.atdd.noaa.gov/pub/GCOS/WMO-Normals/RA-VI/IY/16164.TXT> | 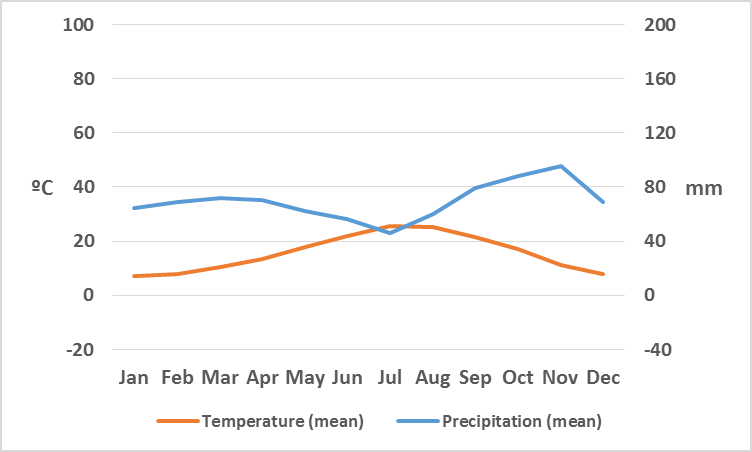 |
| Sampling site: IC  Meteorological station:  Decimomannu, Italy  39º 21' N 008º 58' E, 28 m  <ftp://ftp.atdd.noaa.gov/pub/GCOS/WMO-Normals/RA-VI/IY/16546.TXT> | 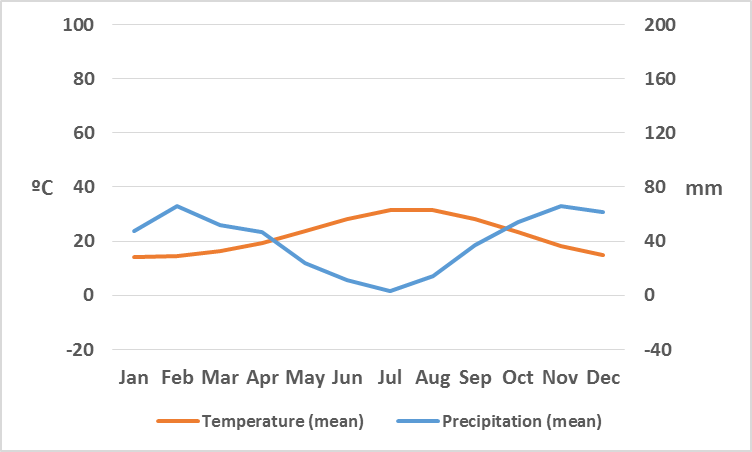 |
| Sampling site: IR  Meteorological station:  Pratica di Mare, Italy  41º 39' N 012º 27' E, 21 m  <ftp://ftp.atdd.noaa.gov/pub/GCOS/WMO-Normals/RA-VI/IY/16245.TXT> | 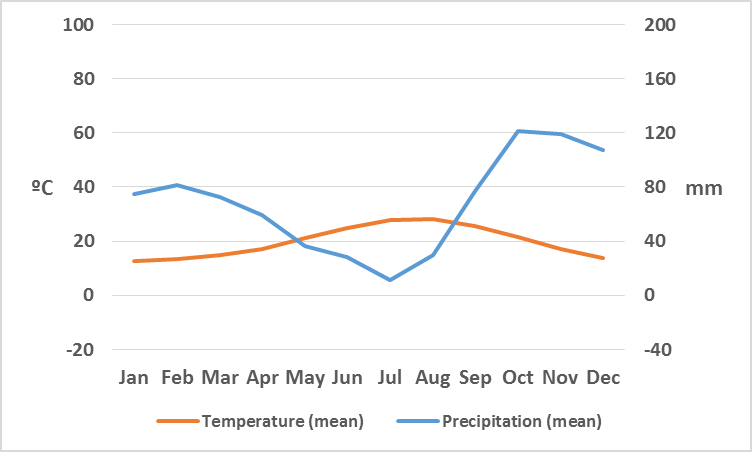 |
| Sampling site: GA  Meteorological station:  Athens Observatory, Greece  37º 58' N 023º 43' E, 107 m  <ftp://ftp.atdd.noaa.gov/pub/GCOS/WMO-Normals/RA-VI/GR/16714.TXT> | 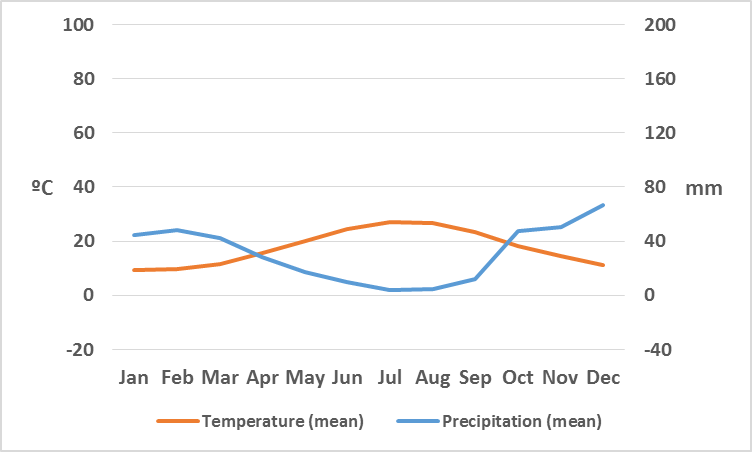 |
| Sampling site: GS  Meteorological station:  Thessaloniki/Mikta, Greece  40º 31' N 022º 58' E, 4 m  <ftp://ftp.atdd.noaa.gov/pub/GCOS/WMO-Normals/RA-VI/GR/16622.TXT> | 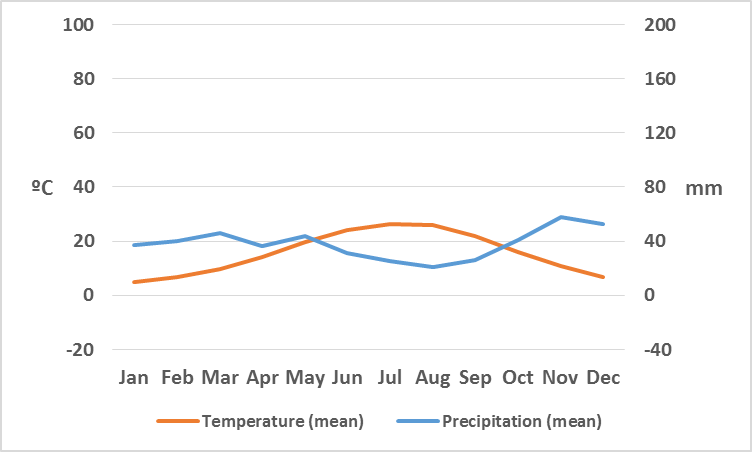 |
| Sampling site: TC  Meteorological station:  Canakkale, Turkey  40º 08' N 026º 24' E, 3 m  <ftp://ftp.atdd.noaa.gov/pub/GCOS/WMO-Normals/RA-VI/TU/17112.TXT> | 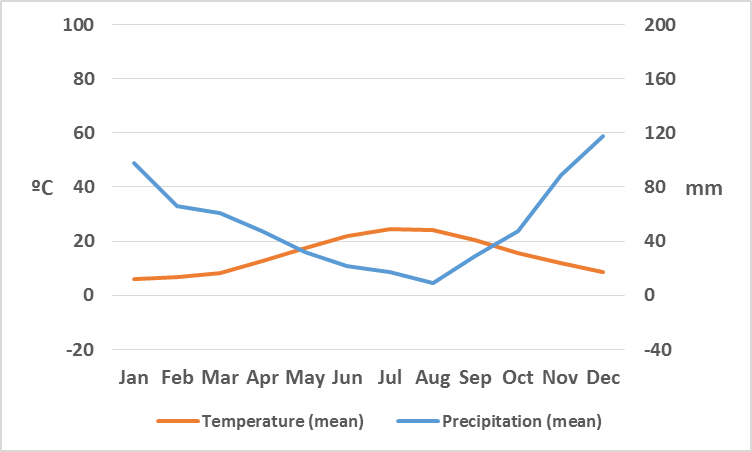 |
| Sampling site: TI  Meteorological station:  Izmir, Turkey  38º 26' N 027º 10' E, 25 m  <ftp://ftp.atdd.noaa.gov/pub/GCOS/WMO-Normals/RA-VI/TU/17220.TXT> | 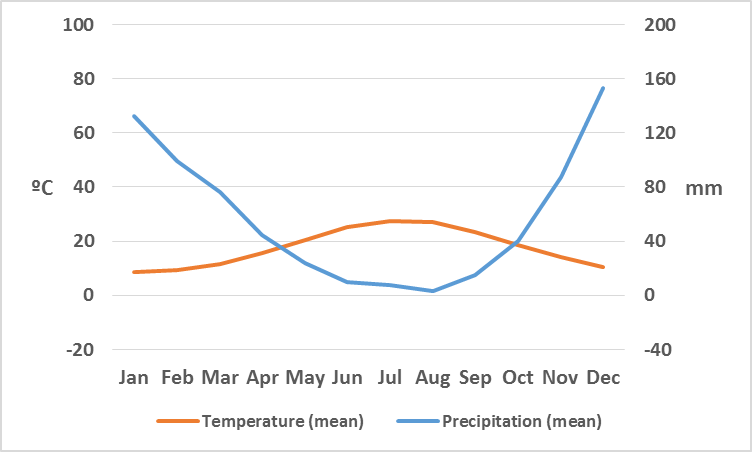 |

National Oceanic and Atmospheric Administration (2015). *Global Climate Normals (1961-1990) by World Meteorological Organization (WMO) Regional Association (RA)* [Online]. Available: <https://www.ncdc.noaa.gov/wdcmet/data-access-search-viewer-tools/global-climate-normals-1961-1990> [Accessed November 27th, 2015].

World Meteorological Organization (2015). *Statistical depictions of climate* [Online]. Available: <http://www.wmo.int/pages/themes/climate/statistical_depictions_of_climate.php> [Accessed November 27th, 2015].
